# Broadband Infrared Absorption Features of Metasurfaces Constructed with a Titanium–Dielectric–Titanium Array Architecture

**DOI:** 10.3390/nano16080497

**Published:** 2026-04-21

**Authors:** Chuang Zhang, Jiaqi Hu, Han Chen, Xuan Shao, Xinzhe Yao, Fangchen You, Haiwei Wang, Xinyu Zhang

**Affiliations:** 1 National Key Laboratory of Science & Technology on Multispectral Information Processing, Huazhong University of Science & Technology, Wuhan 430074, China; m202373645@hust.edu.cn (C.Z.);; 2 School of Artificial Intelligence & Automation, Huazhong University of Science & Technology, Wuhan 430074, China; 3 China Southern Power Grid Energy Storage Co., Ltd., Guangzhou 511400, China; 4 Wuhan National Laboratory for Optoelectronics, Huazhong University of Science & Technology, Wuhan 430074, China

**Keywords:** IR metasurface, surface wavefield resonant excitation, wideband IR absorption, titanium-based nanodisk and nanocylinder

## Abstract

This study proposes an effective method for realizing broadband-infrared (IR)-equivalent absorption using a metasurface constructed by shaping a metal–insulator–metal structure leading to a semi-opened nanocavity. The metasurface architecture is formed according to an optimized structural configuration and mature micro–nano-fabrication flow. Both the surface travelling and localized resonant wavefield accumulation excited by incident lightwaves in a broad wavelength range of 1–14 μm can be efficiently manipulated based on a dipole molecule antenna responding mechanism. An electromagnetic wavefield shielding effect within the semi-opened nanocavity and the standing-wave formation around the metasurface near-field based on an arrayed titanium–dielectric–titanium structure are examined in detail. The measured IR spectral absorption characteristics reveal that the metasurfaces based on an arrayed top titanium cap with the featured dimensions of 2.0 μm and 2.4 μm can be used to achieve an average equivalent IR absorptivity higher than 80% and 82%, respectively, across a broad wavelength range of 1.29–14 μm, which covers the traditional short-, medium- and long-wave IR bands.

## 1. Introduction

Current research and applications demonstrate that metasurfaces, as special functional micro–nano-structures or materials, have exhibited continuously developing potential in a broad field, for instance, antireflection coatings in solar cells [1], as they can remarkably reduce the scattering loss by enabling the efficient capture of both visible beams and earth-emitted infrared (IR) radiation, thereby enhancing the energy collection and utilization efficiencies. In general, the essential principle for achieving highly efficient lightwave manipulation primarily relies on an arrayed micro–nano-configuration for diffractively modulating the electromagnetic response behaviors [2,3]. Recent breakthroughs in typical micro–nano-fabrication and smart lightfield transformation strategies [4,5] have provided some innovative solutions for multi-spectral wavefield manipulation, such as the typical visible and IR target stealth, which is mainly based on still-developing diffractive metasurface technology [6,7,8,9,10]. As demonstrated, a metasurface can be constructed according to a precise arrangement of artificially designed sub-wavelength elements to smartly adjust the lightwave parameters, including the amplitude, phase, polarized state, wavelength, or wave vector [11,12]. By configuring a multi-resonant coupled meta-atom/molecule or -charged cluster as a basic component of the metasurface, the travelling or localized surface electromagnetic resonance can be efficiently excited over a very thin surface architecture across several typical wavebands, such as the visible, IR, terahertz, or even microwave waveband [13,14,15,16]. Since the featured dimensions of the metasurface can be comparable with the wavelength based on the current micro–nano-fabrication approach, the electromagnetic responses can be further dynamically optimized by easily adjusting the geometric parameters of the basic element or functional unit [17,18,19,20]. The special physical mechanism that originates from the localized or traveling surface wavefield resonance [21] therefore facilitates constructing a patterned sub-wavelength lightfield or complicated wavefront [22].

This article addresses the major challenge of constructing two types of metasurfaces to realize a highly efficient broad absorption of incident IR radiation in a near-field fashion based on the sub-wavelength thickness configuration of a dielectric medium sandwiched between two functional titanium films. The work mainly focuses on resolving the problem of achieving broadband IR absorption without obvious Joule heat accumulation, thereby providing key parameter configuration guidance for optimizing the metasurface design in several aspects, including miniaturization, wideband absorption, and ideal environmental adaptability. The basic functional element design originates from the common metal–insulator–metal resonance mechanism, offering a foundational framework for the highly efficient collection of incident IR radiation that exists or is in temporary storage in the near-field region around the metasurface based on a basic dipole net charge oscillation as a molecule antenna. Relatively strong incident radiation in a broad wavelength range spanning from typical short- to long-wave IR can therefore be effectively processed by the metasurface developed using this design.

## 2. Modeling and Simulation

### 2.1. Basic Simulation Setting

In this study, the finite-difference time-domain (FDTD) method based on Lumerical FDTD Solutions 2023 R1(Ansys Inc., Canonsburg, PA, USA) was utilized for the simulation of the model constructed. Its core principle is to replace the first-order partial derivatives of the electromagnetic field components with respect to time and space by central difference quotients, thereby advancing the propagation process of the electromagnetic waves step by step in the time domain. In the spatial domain, the FDTD is employed to discretize the space into cubic cells with variable size, and orthogonally decompose the electric and magnetic fields using Yee cells to realize the discrete solution of Maxwell’s equations. The transmittance and reflectance are then calculated by adding monitors, and thus the absorptance of the metasurface can be derived from a simple relation of A(ω)=1−R(ω)−T(ω). When performing simulations using the Lumerical FDTD Solutions 2023 R1 (Ansys Inc., Canonsburg, PA, USA), a rational setting about several key parameters is crucial to the calculation accuracy and efficiency. In the simulations, the global mesh size is set at about 50 nm, and a local mesh size of about 10 nm is applied to key structural regions (titanium nanodisks/nanocylinders, vanadium dioxide dielectric layer, and titanium film interfaces), to ensure the calculation accuracy. The common periodic boundary conditions are utilized in both the x- and y-directions for simulating the infinite periodic distribution of the metasurface. In addition, the perfectly matched layer boundary conditions are adopted only in z-direction for describing the absorption character of the outgoing electromagnetic waves from the top and bottom of the structural model, so as to avoid electromagnetic wave reflection at the boundaries and thus ensure the accuracy of simulations. The optical constants of the materials employed in the simulations were extracted from the Lumerical FDTD material database and relevant literature [23,24], as shown in Table 1. The temperature was set at room temperature (298 K, 25 °C) during the simulations.

### 2.2. Metasurface with an Arrayed Nanodisk Top

**Figure 1:**
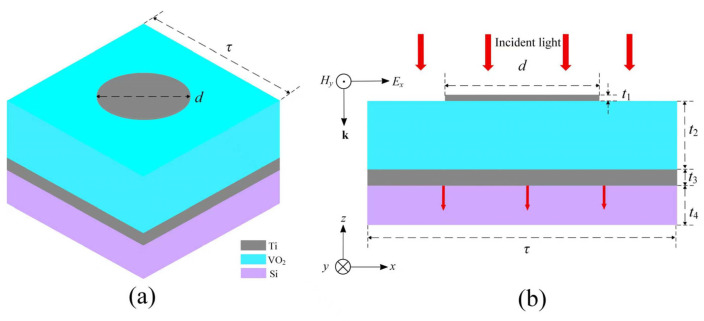
Schematic diagram of a layered configuration of the BIAM proposed. (**a**) A typical 3D viewing of an element and (**b**) a segmented cross-sectional presentation with main structural parameters.

Recently, the approaches for realizing strong broadband IR absorption based on a metasurface configured by an arrayed diffractive structure, are rapidly developed. A functioned diffractive element is generally fabricated using homogeneous material. Titanium exhibits a large imaginary part of the dielectric function across the mid-wave and the long-wave IR region, enabling highly efficient lightwave coupling and absorbing over an ultra-broad wavelength range. Given its moderate and stable refractive index, low extinction coefficient, and high film quality in the infrared band, VO_2_ is employed as the dielectric layer and is in its insulating state at room temperature. For continuously prompting the IR absorption efficiency in a broad wavelength range crossing two or three atmospheric windows, we propose a new broadband IR absorption metasurface (BIAM), which is constructed by sandwiching a single-dielectric VO_2_ layer between an arrayed titanium nanodisk and the same material bottom film over a common silicon wafer, leading to a semi-opened nanocavity with a typical metal–isolator-metal fashion. Generally, the surface of the top titanium nanodisk will rapidly evolve into a dense oxide with a typical thickness of 5–10 nm in air, thereby completely isolating any moisture and further oxygen corrosion. Practically, the titanium material will exhibit ideal reflectivity in a broad IR waveband. Simultaneously, significant thermal conductivity will result in rapid local heat dissipation, reducing the risk of thermal deformation of the thin surface titanium texture. As is known, the featured insulator-to-metal phase transition characteristic of VO_2_ material makes it a unique dynamic medium for achieving some special photonic functions. An obvious alteration in crystal structure can therefore be performed by dynamically modulating the equivalent optical thickness of the MIM architecture, enabling a reset of the radiation absorption efficiency and the lightwave phase away from the top or bottom titanium film of the BIAM. A basic functional element illuminated by incident light, where the IR radiation with electric- and magnetic-component (*E_x_*, *H_y_*) is guided by a wave-vector ***k*** according to a common rectangular coordinate, as shown in Figure 1. The basic structural period *τ* and a single titanium nanodisk diameter *d* are 4 μm and 2 μm, respectively. The thickness of the grey top and bottom titanium films are *t*_1_ = 20 nm and *t*_3_ = 100 nm, and the blue VO_2_ layer thickness *t*_2_ = 1.05 μm. The purple Si substrate is a standard 4-inch single-polished silicon wafer with thickness *t*_4_ = 500 μm.

**Figure 2:**
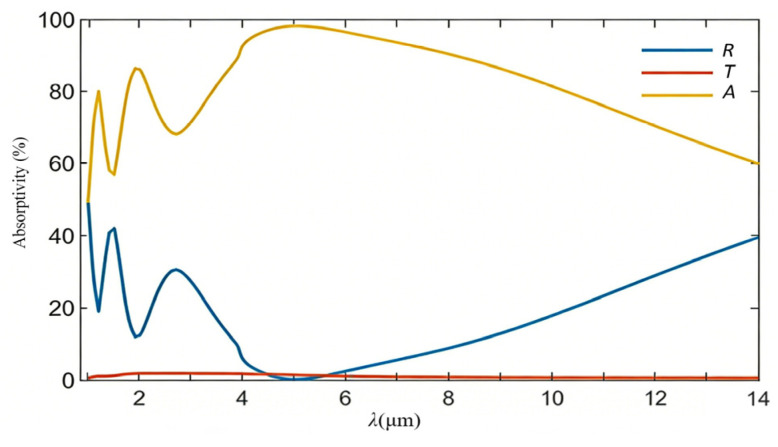
The simulated IR spectral characters of the BIAM including the blue radiation reflection and red transmission and yellow equivalent absorption in a broad wavelength range of 1–14 μm.

The simulated IR spectra are presented in Figure 2, where *R* and *T* express the reflection and transmission spectra of the metasurface proposed, respectively, and *A* is defined as an equivalent absorption spectrum. During simulations, a uniform sampling is conducted at 131 discrete points across a wavelength range of 1–14 μm. The *A* value can be directly calculated by a simple relation, *A*(ω) = 1 − *R*(ω) − *T*(ω), where ω is the angular frequency of incident radiations. As demonstrated, the BIAM already exhibits an average transmittance of about 1% across the entire wavelength range mentioned. The titanium film with a thickness already exceeding the material’s skin depth has functioned as a perfect beam reflective mirror. The absorption spectrum exhibits an initial wave-shaped profile with two distinct peaks at about 1.2 μm and 1.9 μm in a short waveband of 1–4.7 μm, which should be governed by the featured radiation reflection of the metasurface constructed, and then a gradually falling trend from a peak of about 98% at about 5 μm in a long waveband of 5–14 μm. An average equivalent absorptivity of about 82% thus indicates an almost complete capture of incident IR radiations, even those having a minimum value of about 66% at 14 μm. This phenomenon can be interpreted by effectively establishing stable resonant near-field standing waves upon the incident surface of the metasurface already presenting the longest extending distance of around 260 μm from the top of the BIAM, as demonstrated by two right subfigures. As is known, the near-field lightwaves must surround the charged-beam manipulation architecture, which cannot be emitted as classic electromagnetic radiation detected by common sensors, leading to a particular IR radiation-equivalent absorption or temporary storage mechanism without an observable increase in Joule heat.

**Figure 3:**
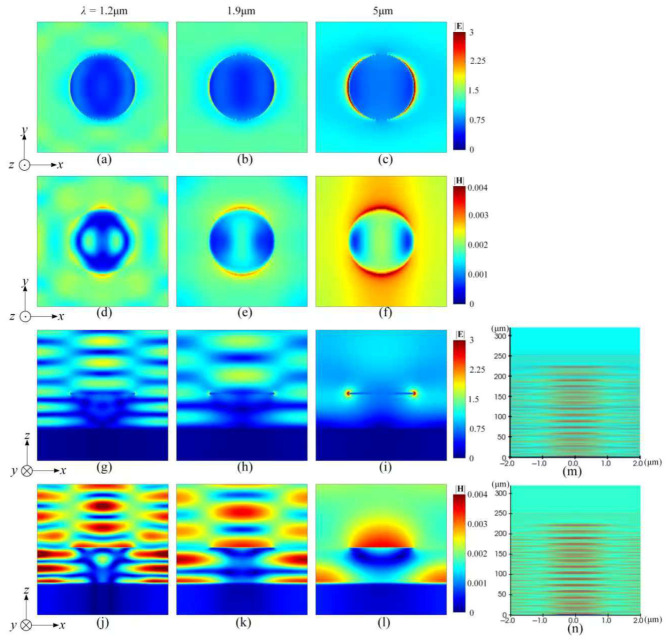
Typical electric- and magnetic-field distributions over both the *xy*-plane and *xz*-plane at several featured wavelengths selected according to the simulations mentioned above, where the transverse amplitude distributions are indicated according to a color bar varying from dark blue for a minimum value of 0 to dark red for varying maximum values. (**a**) 1.2 μm *xy*-plane electric field distribution; (**b**) 1.9 μm *xy*-plane electric field distribution; (**c**) 5 μm *xy*-plane electric field distribution; (**d**) 1.2 μm *xy*-plane magnetic field distribution; (**e**) 1.9 μm *xy*-plane magnetic field distribution; (**f**) 5 μm *xy*-plane magnetic field distribution; (**g**) 1.2 μm *xz*-plane electric field distribution; (**h**) 1.9 μm *xz*-plane electric field distribution; (**i**) 5 μm *xz*-plane electric field distribution; (**j**) 1.2 μm *xz*-plane magnetic field distribution; (**k**) 1.9 μm *xz*-plane magnetic field distribution; (**l**) 5 μm *xz*-plane magnetic field distribution; (**m**) Near-field electric field distribution in the *xz* plane; (**n**) Near-field magnetic field distribution in the *xz* plane.

The typical spatial electric- and magnetic-field distributions over both the *xy*-plane and *xz*-plane at three featured wavelengths of 1.2 μm, 1.9 μm, and 5 μm are exhibited in Figure 3. The typical transverse electric-field distributions are presented in Figure 3a–c and Figure 3g–i, and the correlated magnetic fields over the same planes are shown in Figure 3d–f and Figure 3j–l, respectively. In general, the electric-field emission can be attributed to a typical electric-dipole oscillation of a pair of negative and positive net charge clusters accumulated over two opposite side edges of a single titanium nanodisk along the *x*-direction, which can also be considered a type of molecule antenna. By gradually increasing the measuring wavelength from an initial 1.2 μm to 5 μm, the transverse electric-field amplitude around a single nanodisk will gradually decrease, as can be observed from similar locations. As presented in Figure 3a–c, an obvious variance trend of the spatial electric-field excited around a single nanodisk is indicated by a colored sequence trending initially from dark green, with an approximate value of 1.66 at 1.2 μm, to light blue (0.95 at 1.9 μm) and then to blue (0.82 at 5 μm) according to the color bar attached. However, the spatial electric fields closely nearing the side edge of the nanodisk present a variance trend with a colored sequence changing from its initial light blue color, having an approximate value of 1.02 at 1.2 μm, to light yellow (1.87 at 1.9 μm) and then to dark red (3 at 5 μm). Noting that the crescent morphology and its featured width of the dark red electric-field distribution must be closely related to the arranging patterns of both the positive and negative net charges over two side edges of a single nanodisk. The above characteristics are basically consistent with a traditional standing-wave lightfield formation through a superposition of the incident lightwaves and the lightfield emitted from a dipole antenna, while gradually decreasing the oscillating or emitting frequency. As shown in Figure 3d–f, the correlated oscillating magnetic fields stimulated around the same nanodisk can also be evaluated based on a patterned spatial distribution along the *y*-direction, which must be perpendicular to the emitted electric fields as the source originated from the same electric-dipole molecule antenna mentioned. By increasing the measuring wavelength, the average amplitude will be gradually enhanced from its initial yellow color, with an average value of 0.0026, to orange (0.0029) and then red (0.0038). It can be expected that the accumulated spatial magnetic-field amplitude presenting a similar initial crescent appearance will be rapidly attenuated while gradually leaving the nanodisk along the *y*-direction.

As shown in Figure 3g–i, an obvious electric-field shielding (EFS) within a semi-opened nanocavity with an extremely weak residual electric field colored by dark blue, can be clearly observed. A transverse electric-field amplitude within the nanocavity will be slightly weakened while gradually increasing the wavelength of incident lightwaves, which is distinctly indicated by the pseudo-color variance from an initial dark blue color at 1.2 μm or 1.9 μm to slightly faded blue at 5 μm. An obvious near-field standing-wave pattern upon the top of the nanodisk and the bottom titanium film of the BIAM, which is directly exposed to the incident lightwaves through a VO_2_ layer, can be clearly observed. It should be attributed to a strong spatial interference of incident lightwaves and the emitted near-field lightwaves from densely distributed molecule antenna over the surface of the top and bottom titanium film of the metasurface. The accumulating dipole-type oscillation should be excited by both the surface plasmon polaritons (SPPs) originated from the net charge rearrangement over the surface of the bottom titanium film, and the localized surface plasmas (LSPs) originated from both the positive and negative net charges accumulated and then attenuated over two side edges of the nanodisk.

As presented, there exists a phase difference π between two segmented standing-wave fields upon the nanocavity and the exposed bottom titanium. The maximum amplitude and the full width at half maximum (FWH) of the standing-wave fields already exhibit an opposite variance trend, while the measuring wavelength increases. The thin and dense interference stripes will arise at short wavelengths and basically exhibit an intensity sequence of 1.2 μm@yellow, 1.9 μm@light yellow, and 5 μm@light blue, but indirectly exhibiting the largest accumulating extent of the net charges as indicated by the dark red color with an amplitude of 3. The correlated spatial magnetic fields perpendicular to the electric fields along the *z*-direction also demonstrate a similar standing-wave appearance over the BIAM, and then a strong electromagnetic shielding within the semi-opened nanocavity indicated by dark blue, as shown in Figure 3j–l. It should be noted that the patterned electric- and magnetic-field distribution formed upon the metasurface indicates that the frequency and amplitude (intensity) of incident lightwaves already match the resonant condition of the designed periodic nanostructure. The incident IR energy will therefore be highly localized within the near-field region, which will rapidly decay as the distance gradually increases and completely vanish after the viewing distance exceeds about 200 μm. The absorption characteristics in the 1–4.7 μm range are mainly governed by the LSPR of the titanium nanorods, which act as electric-dipole antennas. The strong broadband absorption observed from 5 to 14 μm arises from coupled SPPs excited at the titanium–dielectric interfaces, together with stable near-field standing waves established above the metasurface. Meanwhile, the semi-open nanocavity enables efficient electromagnetic field confinement, which further traps incident energy and broadens the spectral response. By increasing the wavelength, the electric-dipole mode is gradually suppressed, while the cavity-supported standing-wave mode is strengthened and ultimately becomes dominant.

**Figure 4:**
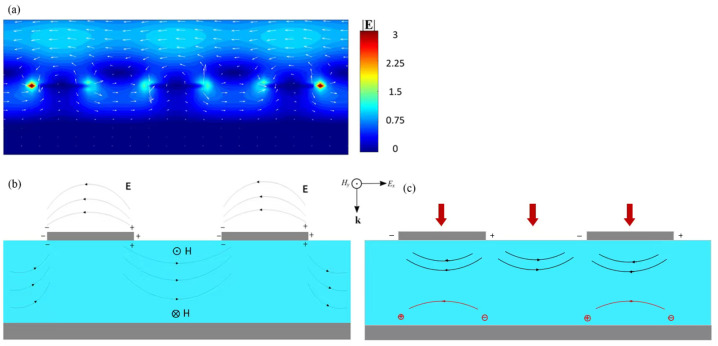
A transverse spatial electromagnetic-field morphology corresponding to only several sets of dipole net charge clusters selected as an essential nano-electronic architecture of the BIAM proposed. (**a**) Detailed spatial electric-field vector distribution stimulated by the incident lightwaves with a central wavelength of 5 μm. (**b**) A typical wave-shaped spatial electric-field **E** with opposite direction upon the nanodisks and in VO_2_ layer, which are tightly confined by the positive and negative net charge clusters accumulated over two opposite side edges of each nanodisk, and the perpendicular spatial magnetic-field **H** coupled with the local electric-field is mainly compressed in VO_2_ medium and also existing in the near-field region upon the BIAM. (**c**) Upon irradiation by incident light, typical localized electric field generated in the dielectric layer originated from the electric-dipole moments excited over the top titanium cap and the surface of the bottom titanium film, respectively.

Figure 4 presents a transverse spatial electromagnetic-field morphology corresponding to several sets of dipole net charge clusters selected as an essential nano-electronic architecture of the BIAM. A patterned arrangement of the spatial electric-field vector around three adjacent nanodisks originated from the incident lightwaves with a central wavelength of 5 μm is shown in Figure 4a. Generally, the strongest near-field electric field correlates closely with the negative and positive net charge clusters, which is indicated by dark red with a value of 3 and is located at the rightmost and leftmost side edges of the three nanodisks. In addition, the accumulated density of the electric fields correlated closely with the net charge clusters is relatively weak, as indicated by light blue regions with a value of about 1.12. This could be attributed to the destructive superposition of the spatial electric fields with varying directions originating from the net charge clusters inducted over the nanodisks and also the surface of the bottom titanium film, as exhibited in the subfigure. A compound circular spatial electric-field with several internal periodic local electric-field patterns around each nanodisk is thus diagramed by densely arranged white arrows.

Figure 4b presents a detailed distribution of both the correlated electric and magnetic fields and the net charge clusters accumulated at two side edges of each nanodisk. Generally, the relatively strong spatial electric-field will be compressed or dispersed over the side edge of the top titanium nanodisk along the x-direction, respectively. By satisfying the resonant condition, the surface “free electrons” of the nanodisk will be transiently accumulated over its side edge, excited by incident lightwaves with the necessary frequency and momentum. Furthermore, the electric-field lines can also be depicted between adjacent nanodisks, indicating that the adjacent individual nanocavities can be correlated with magnetic-field excitation. The electromagnetic field distributions corresponding to top titanium nanodisks are thus predominantly governed by the LSPs. In addition, the phenomenon of the electromagnetic field being obviously weakened at the interface between VO_2_ layer and the bottom titanium is primarily determined by the SPPs over the surface of the bottom titanium film.

### 2.3. Metasurface Based on an Arrayed Multi-Dielectric Nanocylinder

**Figure 5:**
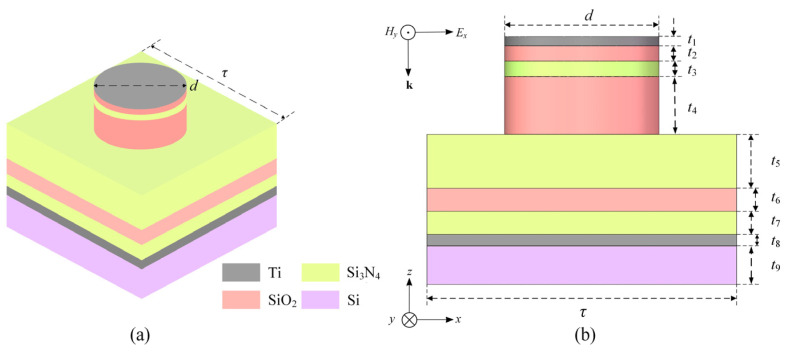
Schematic diagram of the new metasurface formed by coupling an arrayed multi-dielectric nanocylinder with a composite film system formed over a common silicon substrate. (**a**) A 3D viewing including a single multi-dielectric nanocylinder and (**b**) a segmented cross-sectional presentation of an element in the new BIAM proposed.

To further improve the IR absorption efficiency of the BIAM in the long wavelength region of 5–14 μm, another metasurface is constructed by integrating an arrayed multi-dielectric nanocylinder with a bottom titanium film already covered by a layered medium film system. Considering that SiO_2_ and Si_3_N_4_ materials exhibit a low absorption loss and an extremely high transmittance across the mid-wave and long-wave IR regions, they can be used to maximize the light energy coupling through a semi-open cavity, ensuring a high absorption efficiency of the architecture. A basic nanocylinder with a diameter *d* is constructed by attaching a layered dielectric film in an arranging sequence consisting of a top SiO_2_ film with a thickness *t*_2_ and an intermedium Si_3_N_4_ film with a thickness *t*_3_ and then a thick SiO_2_ film with a thickness *t*_4_ below the top titanium nanodisk with a thickness *t*_1_. The operation means that a thin titanium nanodisk mentioned above is completely replaced by a nanocylinder. In addition, a single-dielectric VO_2_ layer coated over the bottom titanium film in the BIAM above is also replaced by a composite film system including a thick Si_3_N_4_ film with a thickness *t*_5_ followed by a SiO_2_ film with a thickness *t*_6_ and another Si_3_N_4_ film with a thickness *t*_7_ also upon a titanium film with a thickness *t*_8_, which are fabricated over a silicon substrate with a thickness *t*_9_. The new BIAM can thus be formed through tightly coupling the nanocylinder and the composite structure, as shown in Figure 5. The basic parameter configuration is as follows: the top nanocylinder diameter *d* and its arranging period *τ* being 2 μm and 4 μm, respectively, the top and bottom titanium film being *t*_1_ = 50 nm and *t*_8_ = 100 nm, the dielectric film being {*t*_2_ = 200 nm, *t*_3_ = 200 nm, *t*_4_ = 750 nm, *t*_5_ = 700 nm, *t*_6_ = 300 nm, *t*_7_ = 300 nm}, and the Si substrate being *t*_9_ = 500 μm.

**Figure 6:**
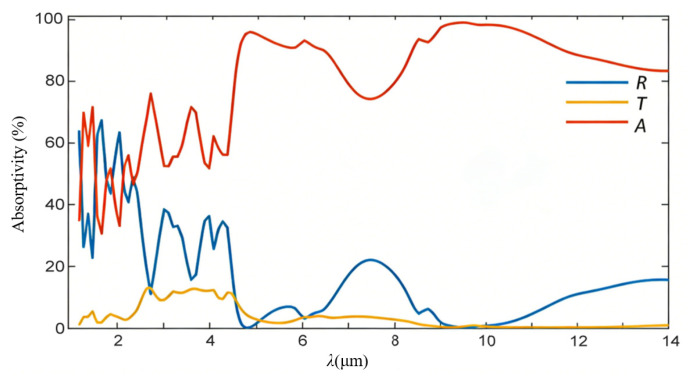
IR spectra of the proposed metasurface with obviously improved IR characteristics in the long wavelength region of 5–14 μm.

As shown in Figure 6, an average equivalent absorptivity exceeding 80% across a wavelength range of 1–14 μm can be achieved according to the simulations. As the wavelength is less than 4.7 μm, both the reflection and transmission curves, indicated by blue and brown, show slightly worse performance than those shown in Figure 2. A similar variance trend in the equivalent IR absorption indicated by red can also thus be observed, which should be attributed to a weakened action of both the electromagnetic wavefield shielding of the similar semi-opened nanocavity and the spatial near-field standing waves constructed upon an arrayed top nanocylinder cap and the bottom titanium film directly exposed to the incident radiations through a multi-dielectric film system. Of particular note is that the refractive-index contrast between the multilayer dielectric structures facilitates strong electromagnetic field redistribution and localized field enhancement inside the cavity, suppresses energy leakage, and strengthens the near-field standing-wave effect. The synergistic effect of these two mechanisms significantly boosts absorption performance across the long-wavelength region of 5–14 μm, achieving an average absorptivity of approximately 88% within this band. This constitutes a distinct improvement relative to the results shown in Figure 2. Detailed spatial distributions of the electric and magnetic fields are analyzed and discussed in the following sections.

**Figure 7:**
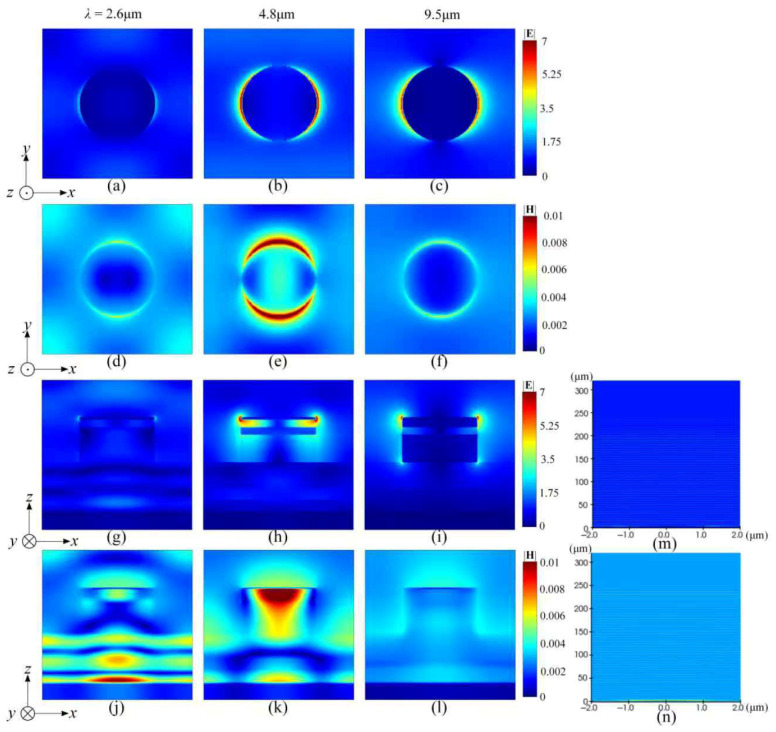
Typical spatial electric- and magnetic-field distribution excited by incident IR radiations with varying featured wavelengths over both the *xy*-plane and *xz*-plane. (**a**) 2.6 μm *xy*-plane electric field distribution; (**b**) 4.8 μm *xy*-plane electric field distribution; (**c**) 9.5 μm *xy*-plane electric field distribution; (**d**) 2.6 μm *xy*-plane magnetic field distribution; (**e**) 4.8 μm *xy*-plane magnetic field distribution; (**f**) 9.5 μm *xy*-plane magnetic field distribution; (**g**) 2.6 μm *xz*-plane electric field distribution; (**h**) 4.8 μm *xz*-plane electric field distribution; (**i**) 9.5 μm *xz*-plane electric field distribution; (**j**) 2.6 μm *xz*-plane magnetic field distribution; (**k**) 4.8 μm *xz*-plane magnetic field distribution; (**l**) 9.5 μm *xz*-plane magnetic field distribution; (**m**) Near-field electric field distribution in the *xz* plane; (**n**) Near-field magnetic field distribution in the *xz* plane.

The typical spatial electric- and magnetic-field distribution over both the xz-plane and xy-plane at different featured wavelengths of 2.6 μm, 4.8 μm, and 9.5 μm, are exhibited in Figure 7. The typical transverse electric fields are presented in Figure 7a–c and Figure 7g–i, and the corresponding magnetic fields in Figure 7d–f and Figure 7j–l, respectively. In general, the emitted electric fields demonstrate a similar distribution and variance trend, as shown in Figure 3. In addition, a similar near-field distance of less than 250 μm can also be viewed in the right subfigure. A pair of negative and positive net charge clusters accumulated over two opposite side edges of a top titanium cap of a single nanocylinder along the x-direction can also be viewed as a molecule antenna. While the measuring wavelength is gradually increased from the initial 2.6 μm to 9.5 μm, the electric-field amplitude (intensity) around a single-molecule antenna is also gradually increased, as can be observed from similar locations. The variance is clearly indicated by a sequence trending initially from light blue, with an approximate value of 2.3 at 2.6 μm, to dark red with a value of 7 at 4.8 μm (but presenting the strongest spatial electric-field excitation) and continuing in dark red with a value of 6.1 at 9.5 μm (with a slightly widened crescent electric-field pattern), as presented in Figure 7a–c.

As shown in Figure 7d–f, the correlated oscillating magnetic fields stimulated around the same nanocylinder can also be recognized according to the patterned spatial distribution along the y-direction, which is also perpendicular to the excited electric fields. By increasing the measuring wavelength, its average amplitude (intensity) will gradually shift from an initial light blue color with an approximate average value of 0.0036 to dark red yellow with a localized maximum value of 0.01 (indicating the strongest magnetic-field emission) and then to light blue with a value of 0.0038. The accumulated spatial magnetic-field amplitude (intensity) presenting a similar initial crescent appearance will be rapidly attenuated, while gradually leaving the nanocylinder along the y-direction. As shown in Figure 7g–i, an obviously weakened electric-field shielding (EFS) within a semi-opened nanocavity compared to that shown in Figure 3, can be observed. The electric-field amplitude (intensity) already intruding into the nanocavity, which demonstrates an transverse amplitude (intensity) sequency of {in the top SiO_2_ dielectric film} > {in the second SiO_2_ dielectric film} >> {in the third or bottom SiO_2_ dielectric film} and existing in both the Si_3_N_4_ dielectric film being very weak, will be increased to the maximum at 4.8 μm, which is obviously stronger than that shown in Figure 3i corresponding to the wavelength of 5 μm. A near-field standing-wave upon the top nanocylinder cap of the BIAM can also be expected; however, it presents a longer wavelength than those shown in Figure 3. These waves are also attributed to a strong spatial interference between incident lightwaves and the re-emitted near-field lightwaves from densely distributed molecule antennas. This interference originates from both the surface plasmon polaritons (SPPs) produced by the net charge rearrangement over the bottom titanium film, and the localized surface plasmas (LSPs) produced by a combination of positive and negative net charges accumulated and then attenuated over two side edges of the nanocylinder. In addition, there also exists a phase difference π between two segmented standing-wave fields upon the nanocylinder and the exposed bottom titanium surface. The relatively wide and sparse interference stripes will arise at short wavelengths. The correlated spatial magnetic fields perpendicular to the electric fields along the z-direction also demonstrate a similar standing-wave fashion on the metasurface, exhibiting a weak electromagnetic shielding within a semi-opened nanocavity, as shown in Figure 7j–l. It should be noted that the patterned electromagnetic wavefield distribution formed upon the metasurface indicates that the frequency and intensity of incident lightwaves already match the resonant condition. The incident IR energy will thus be highly localized within the near-field region and then rapidly decay, while gradually increasing the distance and thus completely vanishing after the viewing distance exceeds about 250 μm.

**Figure 8:**
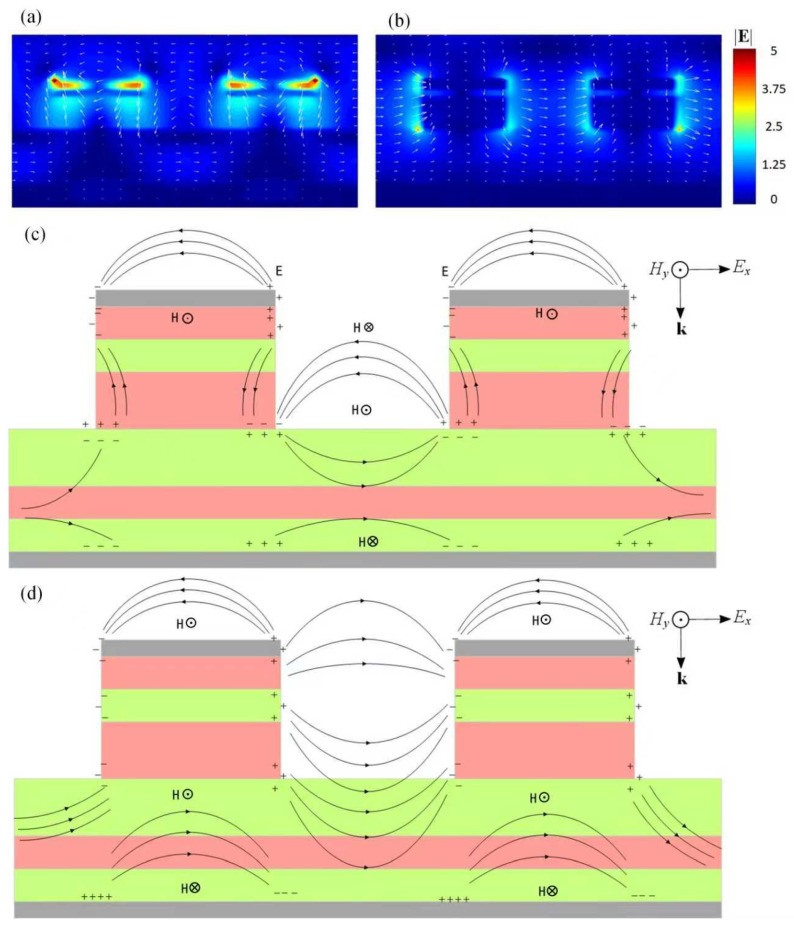
Both the spatial net charge and electromagnetic field distributions excited by IR radiation with varying wavelengths. The arrows represent the direction of the electric field. (**a**,**b**) Patterned spatial electric-field vector arrangement originated from a couple of negative and positive net charges located at two opposite side edges that are excited by incident radiation with the central wavelength of 4.8 μm and 9.9 μm, respectively. (**c**,**d**) Typical transverse spatial electromagnetic field distribution at 4.8 μm without coupling between adjacent nanocylinders and at 9.5 μm with an obvious influence from the coupling between charged nanocylinders.

A transverse spatial electromagnetic-field morphology corresponding to two sets of dipole net charges, as well as to the essential nano-electronic architecture of the BIAM proposed, is shown in Figure 8. Compared to Figure 4, a widened net charge distribution over two side edges of the top titanium is clearly visible. In addition, relatively weak net charge distributions over the two bottom side edges of a single nanocylinder attributed to the excited LSPs, and also over the bottom titanium film originated from the excited SPPs, are labeled. Two patterned arrangements of the spatial electric-field vector around two adjacent nanocylinders, which are stimulated by the incident lightwaves with a central wavelength of 4.8 μm and 9.9 μm, respectively, are shown in Figure 8a. The layered distribution of the electric-field already intruding into the structural region sandwiched by the top and bottom titanium films, and further presenting a stronger invasion at 4.8 μm than at 9.9 μm, obviously enhances an equivalent absorption of the incident IR radiations via the metasurface configurated using an arrayed multi-dielectric nanocylinder. Figure 8d shows the distribution of the correlated electric and magnetic fields in detail and the net charge clusters accumulated at two side edges of each nanocylinder, including the two opposite bottom side edges; it also shows the bottom titanium film albeit not considering the coupling between adjacent nanocylinders. A further enhanced electromagnetic oscillation can be observed compared to that shown in Figure 4 after considering the coupling between adjacent nanocylinders. In conclusion, the electromagnetic wavefield redistribution of the proposed metasurface are predominantly governed by both the LSPs excited by the top titanium caps and the SPPs over the bottom titanium film.

**Figure 9:**
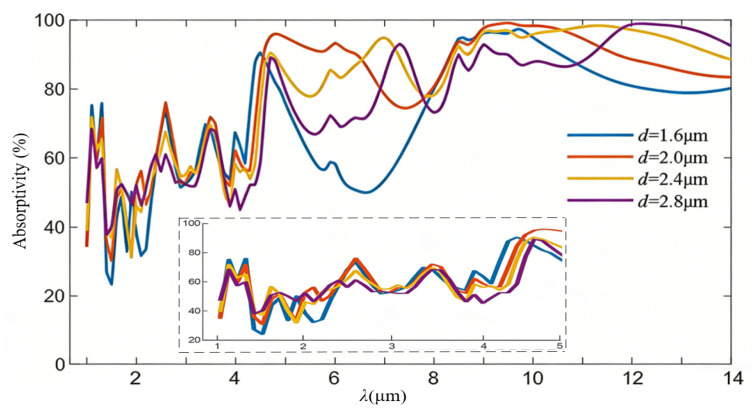
Equivalent IR absorption spectra of the BIAM based on an arrayed nanocylinder architecture with varying top diameter *d*.

The spectral IR absorption in the wavelength range of 1–14 μm through varying the structural configuration, including the top titanium cap diameter *d* and its thickness *t*, are given below. As presented in Figure 9, the equivalent IR absorptivity already exhibits a similar variance trend in the long wavelength region of 5–14 μm by selecting a single cap diameter of 1.6 μm, 2 μm, 2.4 μm, and 2.8 μm, respectively. Increasing *d* redshifts the fundamental dipole resonance of the titanium nanostructures, modulates the equivalent impedance of the metasurface, and reshapes the cavity-mode distribution in the semi-open nanocavity. This consequently directly tailors the coupling efficiency of incident light at different wavelengths, ultimately giving rise to the observed spectral shifts and evolution of broadband absorption. The proposed BIAM exhibits an obvious increase in the average absorptivity in the long wavelength region above. It should be noted that the average IR absorptivity of 1–4.7 μm in the short wavelength region is obviously decreased, which is also presented in the subfigure surrounded by a dashed box. Details of the average absorptivity and spectral peak values are listed in Table 2. Consequently, the top titanium cap diameter should be selected in the 2–2.4 μm range.

**Figure 10:**
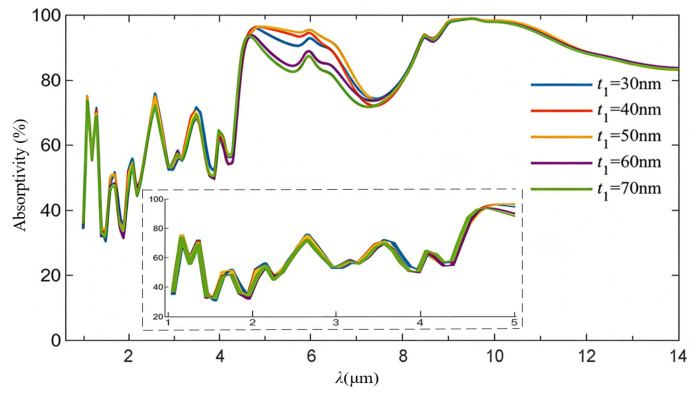
Equivalent IR absorption spectra of the BIAM with varying top titanium cap thicknesses.

The spectral IR absorption in the same wavelength range mentioned above achieved by varying the top titanium cap thickness of a single nanocylinder is demonstrated in Figure 10. Configuring the top titanium thickness in the 30–70 nm range with an interval of 10 nm, the equivalent IR absorptivity exhibits an almost identical variance trend in the long wavelength region of 5–14 μm. An obvious increase in the average absorptivity in the aforementioned long wavelength region can thus be expected, which is accompanied by an obvious decrease of 1–4.7 μm in the same short wavelength region, as shown in the subfigure surrounded by a dashed box. Details of the average IR absorptivity and spectral peak values are listed in Table 3. Consequently, the top titanium cap diameter of a single nanocylinder for the proposed BIAM should be selected in the 2–2.4 μm range.

## 3. Fabrication and Measurement

**Figure 11:**
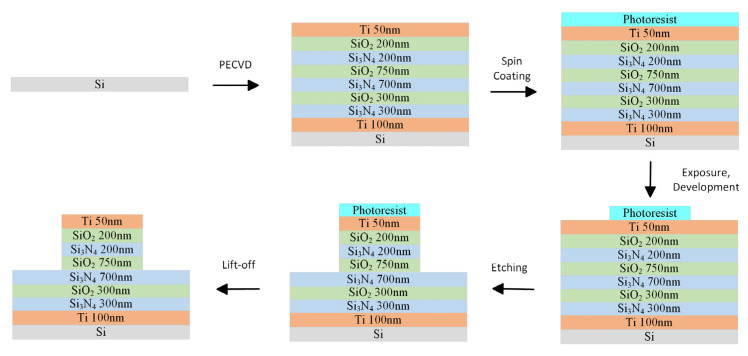
Fabrication process flow diagram.

Based on the simulations with the IR absorption efficiency being improved in the long wavelength region of 5–14 μm, two types of BIAM samples with a single top titanium cap diameter of 2 μm or 2.4 μm are fabricated by employing the mature micro–nano-fabrication technique, mainly including conventional magnetron sputtering, PECVD, UV photolithography, and traditional dry etching. The main fabrication process is illustrated in Figure 11.

**Figure 12:**
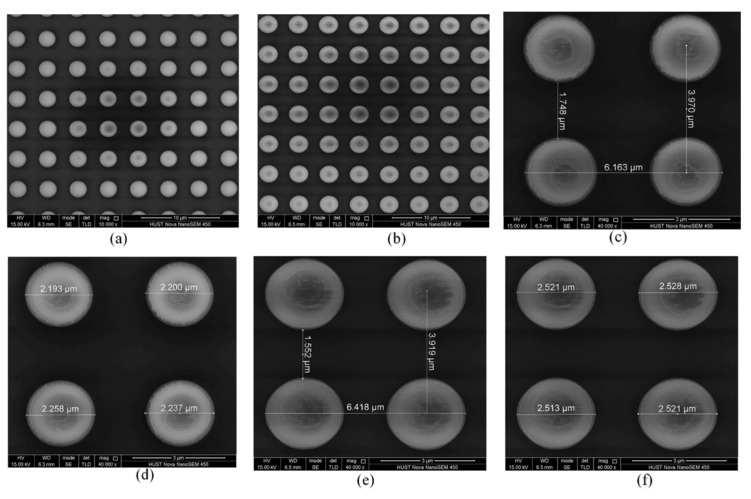
SEM photographs of two BIAM samples formed by an arrayed nanodisk with the required surface structural parameters. (**a**–**f**) SEM images of the typical surface morphology of two arrayed nanodisks with 2 μm and 2.4 μm diameter presented at 10,000× and 40,000× magnification, respectively.

**Figure 13:**
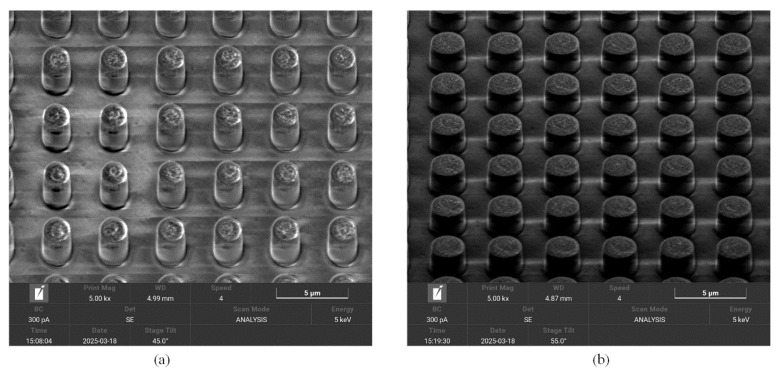
SEM photographs of two BIAM samples formed by shaping an arrayed top nanocylinder with a diameter of 2 μm (**a**) and 2.4 μm (**b**), respectively.

Both the morphology and main structural parameters of the metasurface samples are acquired utilizing the Nova NanoSEM 450 field-emission scanning electron microscope, as presented in Figure 12. Two typical partial surface functional regions of the BIAM samples with a basic top titanium nanodisk of 2 μm and 2.5 μm in diameter, respectively, exhibit a uniform surface structural distribution, as shown in Figure 12a and Figure 12b. Each nanodisk in the different samples presents an approximately circular profile, leading to the following requisite structural configurations: class-1 (the diameter being about 2.2 μm)—the interval size is 1.75 μm and the center-to-center distance is 3.97 μm, as shown in Figure 12c,d; class-2 (the diameter being about 2.4 μm)—the interval size is 1.75 μm and the center-to-center distance is 3.97 μm, as shown in Figure 12e,f. These configurations present a maximum dimensional deviation of about 10% when compared to the simulations. The surface deformation and small structural dimensional deviation likely originate from the small errors during the etching process, which can be remarkably improved by finely optimizing the technological flow. Despite some fabrication errors, the overall structural configuration and the surface morphology of the samples satisfy the fabrication requirement. The two typical partial functional regions of the BIAM samples with a basic titanium nanocylinder of 2 μm and 2.54 μm in diameter also exhibit a uniform top nanocylinder distribution, as shown in Figure 13a and Figure 13b, respectively. It should be noted that each nanocylinder presents an ideal profile with negligible structural deviation according to the measurements.

**Figure 14:**
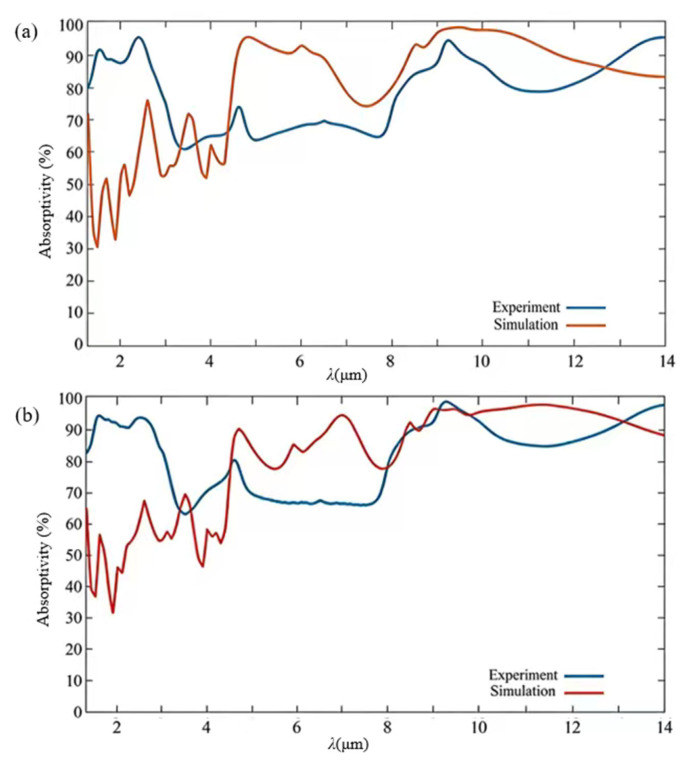
Comparison of the simulated and measured IR absorption spectra of two samples formed using a basic nanocylinder with a diameter of 2 μm (**a**) or 2.4 μm (**b**) in the 1.29–14 μm wavelength range. The IR absorptivity is acquired by directly calculating the reflection and transmission data measured, which demonstrates an obviously improved IR absorption in the long wavelength range of 5–14 μm.

A microscopic IR spectrometer—specifically, Nicolet iN10—is used to measure the samples fabricated by shaping arrayed nanocylinders with diameters of 2.0 μm and 2.4 μm, as shown in Figure 14. According to the mean absolute error (MAE) formula of $MAE=\frac{1}{N}\sum_{i=1}^{N}\left|A_{sim,i}-A_{exp,i}\right|$, the mean absolute errors are calculated to be 19.3% and 14.2%, respectively. The differences could be attributed to several factors such as the micro–nano-fabrication imperfection, the measurement uncertainties, and the adoption of the ideal bulk optical constants in simulations. Practically, the deposited thin films suffer from several subtle deviations due to their dielectric function, basic oxidation, the interface scattering, and other effects, leading to observable differences in absorption intensity. It should be noted that the IR absorptivity is determined by directly calculating from both the reflection and transmission data measured. As shown, the two samples present an average absorptivity higher than 80% and 83% in the 1.29–14 μm wavelength range, as shown in Figure 14a and Figure 14b, respectively. In the short wavelength region of 1.29–4.7 μm, the IR absorptivity presents an obvious difference compared to the simulations. It should be noted that the IR absorptivity exceeds 80% across the whole wavelength range discussed, and although the data values are slightly low compared to the simulations, they still present an improved spectral variance trend, which could be attributed to the obviously enhanced lightwave resonance in the dielectric system sandwiched by the top and bottom titanium films, leading to a semi-opened nanocavity.

**Figure 15:**
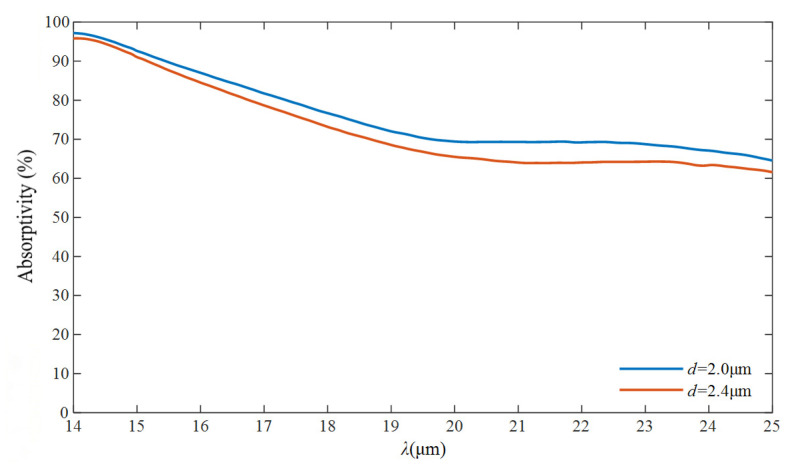
Comparison of the measured IR absorption spectra of two nanocylinder samples with varying diameters in the 14–25 μm wavelength range.

Considering the case that the samples maintain a high absorptivity at 14 μm, the transmission and reflection characteristics of the structures in the long-wave region are subsequently measured using a Fourier transform infrared spectrometer (VERTEX80v FTIR, Bruker Optics GmbH & Co. KG, Ettlingen, Germany). As shown in Figure 15, due to the mismatch between the wavelength and the structural dimensions, the absorptivity exhibits a smooth decreasing trend while increasing the wavelength. Notably, the absorptivity drops to approximately 60% at the central wavelength of 25 μm, while the average absorptivity of the two structures in this band remains at a relatively high level, reaching 75.95% and 72.42%, respectively.

Compared with the reported broadband IR metamaterial absorbers, as shown in Table 4, the titanium–dielectric–titanium array metasurface proposed in this study exhibits some significant advantages in both the absorption performance and fabrication compatibility. In ref. [25], a designed MIM absorber consists of four different chromium nanorings, achieving an average absorption of 94.84% in the range of 800–3000 nm, but its reliance on precisely positioned multi-sized rings will increase the difficulty of photolithography alignment. Ref. [26] presents an MIM absorber that realizes high absorption in the 8–30 μm range; the ultra-small dimensions of its cross-shaped and strip resonators will lead to a demanding fabrication requirement. In ref. [27], the introduced functional materials such as graphene and VO_2_ can be used to realize a tunable metasurface, but they suffer from a relatively low average absorption within the operating band. In contrast, the absorber designed in this work adopts a pure Ti-based symmetric MIM structure with a single-sized periodic array upon the top layer, which can be fabricated using the mature processes including common magnetron sputtering, UV photolithography and dry etching, thus offering a lower cost and a better compatibility based on the standard micro–nano manufacturing. The proposed architecture thus covers a broadband IR range of 1.29–25 μm, which means a wider response range. Regarding absorption performance, the measured average IR absorptivity exceeds ~82% across the entire operating waveband, and the absorptivity in the long-wave region of 8–19 μm exceeds 88%.

## 4. Conclusions

This study develops an effective method for realizing broadband IR absorption using the BIAM, which is constructed by shaping a metal–insulator–metal architecture leading to a semi-opened nanocavity. The metasurface structures are constructed according to an optimized parameter configuration and common micro–nano-fabrication approach. Both the travelling and localized surface wavefield resonant accumulation, excited by the incident lightwaves with the required frequency and momentum in a broad wavelength range of 1–14 μm, can be efficiently manipulated. An electromagnetic wavefield shielding effect within the semi-opened nanocavity and also the patterned standing-wave generation in a near-field region around the metasurfaces are comprehensively explored. The measured IR spectral absorption characteristics reveal that the developed metasurfaces based on an arrayed top titanium cap with the featured dimensions of 2.0 μm and 2.4 μm can be used to achieve an average IR equivalent absorption higher than 80% and 82%, respectively, across a broad wavelength range of 1.29–14 μm, which covers the traditional short-, medium-, and long-wave IR bands.

## Figures and Tables

**Table 1:** Optical parameters (refractive index *n*, extinction coefficient *k*) of materials in wavelength range 1–14 μm.

Material	Refractive Index (*n*)	Extinction Coefficient (*k*)	Applicable Wavelength Range (μm)	Parameter Source
Ti	2.0–4.5	3.0–8.0	1–14	Lumerical FDTD Material Database
VO_2_	2.2–3.0	0.01–0.10	1–14	[24]
SiO_2_	1.44–1.46	<1 × 10^−4^	1–14	Lumerical FDTD Material Database
Si_3_N_4_	2.0–2.1	<5 × 10^−4^	1–14	Lumerical FDTD Material Database
Si	3.45–3.48	<1 × 10^−5^	1–14	Lumerical FDTD Material Database

**Table 2:** Equivalent absorptivity of the metasurface with varying nanocylinder cap diameters.

Cap Diameter (μm)	Average Absorptivity (%)	Spectral Peak Value (%)
1.6	72.22	97.31
2.0	80.70	99.10
2.4	81.18	98.30
2.8	77.38	98.96

**Table 3:** Equivalent absorptivity of the metasurface with varying top titanium cap thicknesses.

Top Titanium Cap Thickness (nm)	Average Absorptivity (%)	Spectral Peak Value (%)
30	88.22	98.81
40	88.45	99.01
50	89.35	98.30
60	85.66	98.57
70	84.49	98.67

**Table 4:** Comparison of our work with reported broadband IR absorbers.

Refs.	Core Structure	Fabrication Cost	Operating Band	Average Absorptivity
[25]	Cr nanoring MIM	High	800–3000 nm	94.8%
[26]	Ti-based multilayer MIM	Medium	8–30 μm	87%
[27]	Gradient metasurface	Medium	0.5–1.5 THz	69%
Our work	Ti-based metasurface MIM	Low	1.29–25 μm	82%

## Data Availability

The original contributions presented in this study are included in the article. Further inquiries can be directed to the corresponding author.
